# An Extremely Rare Manifestation of Multiple Myeloma: An Immunoglobulin D Secreting Testicular Plasmacytoma

**DOI:** 10.7759/cureus.1400

**Published:** 2017-06-27

**Authors:** Ashish Sharma, Tina Binazir, Alexandre Sintow, Chi Chan Lee, Sameer Shaharyar, Jason Tache

**Affiliations:** 1 Internal Medicine, Aventura Hospital and Medical Center; 2 Oncology, Westside Regional Medical Center

**Keywords:** immunoglobulin d, multiple myeloma, testicular plasmacytoma, igd, plasmacytoma

## Abstract

Multiple myelomas (MM) of the immunoglobulin D (IgD) subtype is rare amongst plasma cell malignancies. It can present a diagnostic challenge because of the low amount of immunoglobulin in the serum. The amount of monoclonal (M)-protein is often undetectable on electrophoresis. Historically, survival in these patients was typically shorter compared to the immunoglobulin A (IgA) and immunoglobulin G (IgG) subtypes due to advanced disease upon presentation. With the advent of better diagnostic techniques, the prognosis of this disease is changing. We describe a case of an extramedullary testicular plasmacytoma (EMP) of the IgD subtype as the primary feature of MM, which responded well to novel therapy.

A 72-year-old White male presented to the emergency room with a right testicular mass for three months. He subsequently underwent right radical orchiectomy. Pathology of the specimen revealed plasmacytoid cells positive for cluster of differentiation (CD79a), lambda free light chain, IgD, and BCL-1 (Cyclin D1) on immunochemical stains. Urine and serum immunofixation were positive for monoclonal IgD with lambda light chain specificity and Bence Jones proteinuria. Bone marrow biopsy showed large sheets of plasma cells with greater than 90% cellularity. Flow cytometry displayed atypical plasma cells expressing cluster of differentiation (CD38, CD20, and CD56) with cytoplasm and lambda light chain, approximately 20%, consistent with a plasma cell dyscrasia. Stage 3 IgD lambda multiple myeloma was diagnosed. He received novel treatment with Bortezomib and dexamethasone for three months, followed by Lenalidomide. His performance status and lab data improved significantly. He had progression-free survival (PFS) of approximately three years and remained in complete remission low-dose dose of Lenalidomide daily.

IgD myeloma was considered a diagnostic challenge due to undetectable M-protein levels on serum protein electrophoresis (SPEP). With the advent of serum free light chain assay and serum and cytologic examinations, diagnostic accuracy has significantly improved. The IgD subtype is commonly associated with poor clinical outcomes. However, the use of novel agents and autologous transplant has changed the prognosis of this disease.

## Introduction

Multiple myelomas (MM) is a malignant neoplasm of proliferative plasma cells which accounts for 10% of all hematological cancers and 1% of all cancers [1]. It is characterized by proliferation of a single malignant clonal plasma cell in the bone marrow resulting in an abnormal increase in monoclonal immunoglobulin (Ig) protein. Ninety percent of all myelomas are immunoglobulin G (IgG), immunoglobulin A (IgA) and the light chain with a prevalence of 52%, 21%, and 16% respectively. Immunoglobulin D (IgD) myeloma is relatively uncommon, with a prevalence of 1-2% [2], however, its prevalence has been more notable in East Asian countries.

An abnormal increase in monoclonal Ig causes end-organ damage commonly manifesting as hypercalcemia, renal insufficiency, anemia, and lytic bone lesions. Extramedullary involvement can precede the development of frank myeloma, occur at diagnosis or during the course of the disease [1]. Extramedullary plasmacytoma (EMP) can involve multiple anatomic sites. The head and neck are common with a predilection for the respiratory and gastrointestinal (GI) tract [3-4]. Skin, soft tissue, lymph nodes, liver, ovaries, and testicles are less common sites of involvement [3,5]. Isolated testicular plasmacytoma is considered as an advanced disease upon presentation. Following the first reported case in 1939, less than 51 cases of testicular plasmacytoma have been reported [5]. This case report presents a testicular plasmacytoma of IgD subtype as the primary presenting site of an underlying MM. The patient was treated with right radical orchiectomy, followed by myeloma directed chemotherapy.

## Case presentation

A 72-year-old White male presented to the emergency room with a right testicular mass and fatigue for three months. He had multiple co-morbid conditions including diabetes, hypertension, cardiomyopathy status post automated implantable cardioverter-defibrillator (AICD) and renal insufficiency. He denied fever, back pain, and night sweats. He also denied weight loss, although we do not have a record of his prior weight to compare. His physical examination was unremarkable except for a right testicular mass with mild local tenderness.

**Figure 1:**
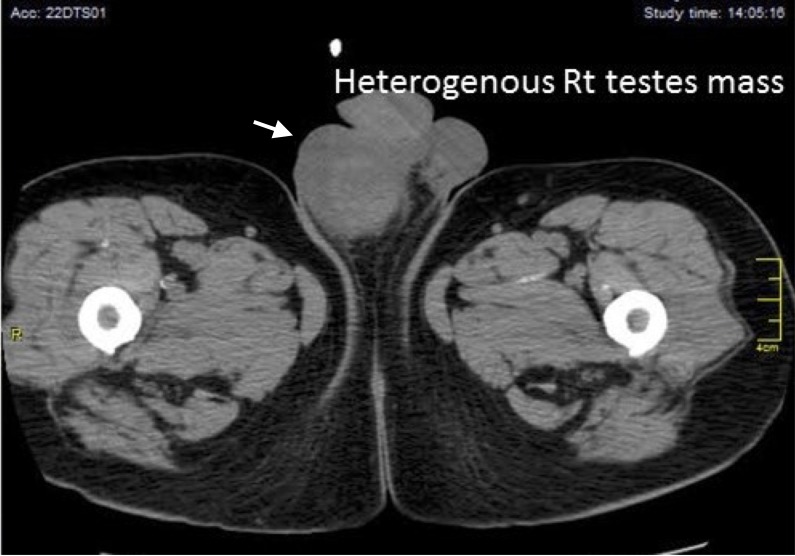
Figure showing the computed tomography (CT) scan of right heterogeneous testicular mass with hyperdense areas

**Figure 2:**
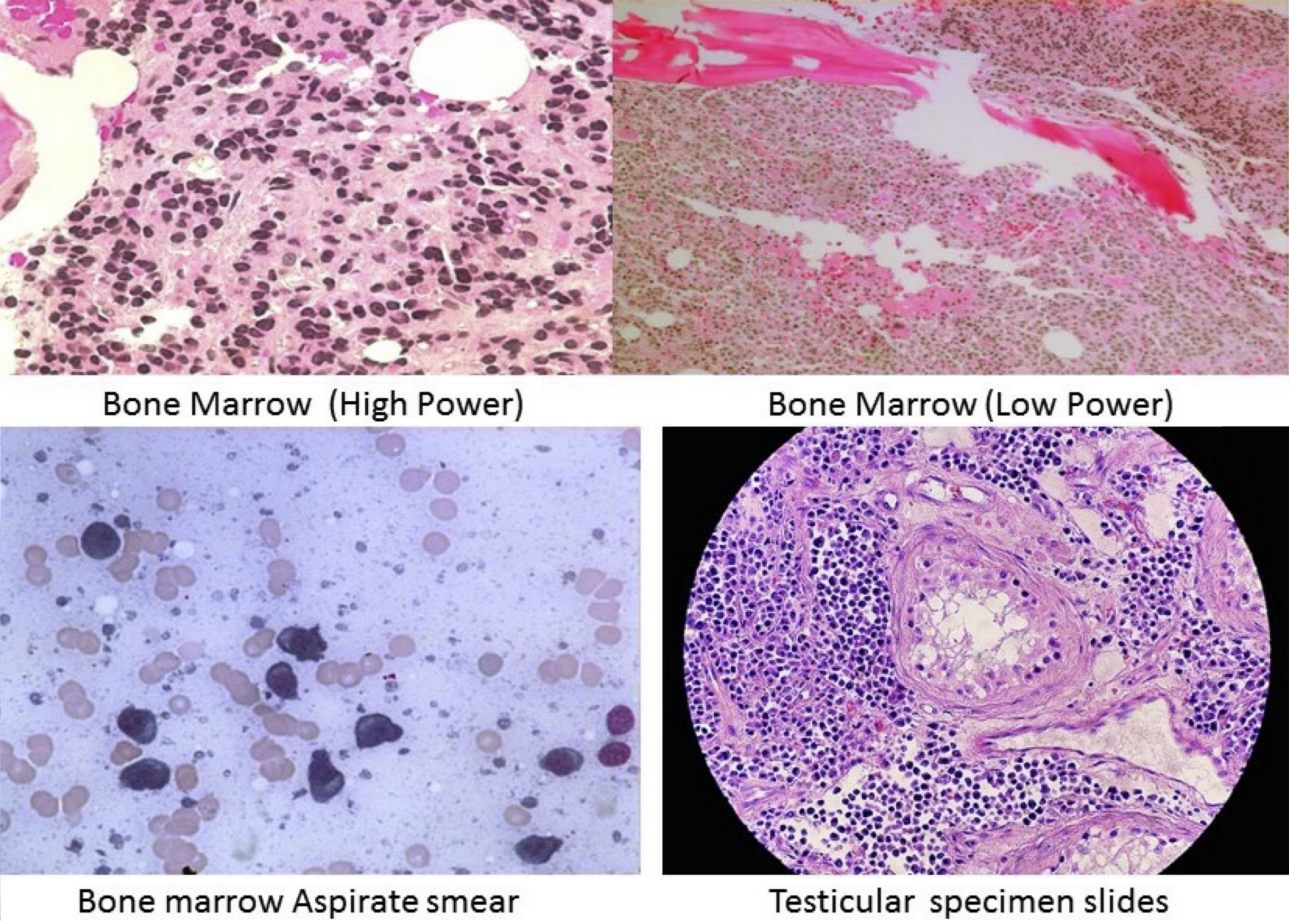
Figure showing the histological slides of bone marrow and testicular specimen showing plasma cells

Initial laboratory studies showed a hemoglobin of 10.5 g/dL, normal white blood cell count (WBC) and platelet count, creatinine 2.59 mg/dL, calcium 15.3mg/dL, parathyroid hormone (PTH) intact 9pg/mL, albumin 4.3g/L, and serum globulin 4g/dL. Serum alpha-fetoprotein and beta- human chorionic gonadotropin (HCG) results were normal. Testicular sonogram and computed tomography (CT) pelvis revealed an enlarged right testicle and multiple hypoechoic lesions with heterogeneous attenuation representing masses (Figure 1). The patient underwent right radical orchiectomy. Pathology of the specimen revealed plasmacytoid cells forming nodules between the seminiferous tubules and extension into the epididymis (Figure 2). The tumor cells showed a high proliferative index. Immunochemical stains were positive for CD79a, lambda free light chain, IgD, BCL-1 and focally epithelial membrane antigen (EMA). Studies were negative for placental alkaline phosphatase (PLAP), kappa light chain, CD30, IgA, immunoglobulin M (IgM) and Epstein-Barr virus-encoded small RNAs (EBER). Flow cytometry of the testicular biopsy showed positive surface lambda light chain restriction, negative cytoplasmic light chain restriction, and a high proliferation index.

Nephelometry showed: IgG 486 mg/dL, IgA 40 mg/dL, and IgM 2 mg/dL with a monoclonal (M) spike of 0.2 g in the beta region and 2.4 g in the gamma region. Urine and serum immunofixation were positive for monoclonal IgD with lambda light chain specificity and Bence Jones proteinuria. Urine protein electrophoresis revealed 7905mg of protein in 24 hours with an M-spike of 83%. Serum IgD was 4210 mg/dL, free lambda chain 6182 mg/dL, with Kappa/lambda < .01 and serum beta-2 microglobulin 7.7 mg/dL [6]. Bone marrow biopsy showed large sheets of plasma cells with greater than 90% cellularity (Figure 2). Flow cytometry results displayed atypical plasma cells with CD38, CD20, and CD56 expression with cytoplasm and lambda light chain, approximately 20%, consistent with a plasma cell dyscrasia. Fluorescence in situ hybridization (FISH) panel revealed an abnormal multiple myeloma panel, positive cytogenetics for cyclin D1/immunoglobulin heavy chain (CCND1/IGH) rearrangement, and loss of tumor protein p53 (TP53). A skeletal survey showed subcentimeter lucencies on the skull and a healed left humerus fracture, but no lytic or blastic lesions. A whole-body positron emission tomography–computed tomography (PET-CT) was unremarkable.

The patient was diagnosed with stage 3 international staging system (ISS), IgD lambda multiple myeloma four months after the onset of symptoms. His performance status was marginal. The selected treatment plan was initiated one month after diagnosis was made. We administered double chemotherapy protocol, as he was not an appropriate candidate for autologous stem cell transplant (ASCT) secondary to age and comorbidities. Bortezomib and Dexamethasone were started. The patient’s performance status improved after three months, and at that time, a once-daily dose of Lenalidomide was added to the regimen. He showed excellent response to the treatment which was reflected in his clinical and biochemical (particularly anemia and metabolic) profiles. Although his renal function did not return to normal, it remained stable without progression. His IgD protein burden decreased from 4210 mg/dL to 20 mg/dL after nine total cycles of combination therapy. Follow-up serum protein electrophoresis (SPEP) and immunofixation studies did not show M-protein, and his repeat bone marrow biopsy and FISH study were also unremarkable. Dexamethasone was gradually tapered within a three to a six-month interval, and he was kept on low dose Lenalidomide daily. On his last follow-up visit in the clinic, Hb was 13 g/dL, white blood cell (WBC) 4.4 k/uL, platelets 107000/mm3, and creatinine was 2.6 mg/dL. He had a progression-free survival (PFS) of approximately three years and remained in complete remission. One year after remission, the patient was admitted to the intensive care unit for respiratory failure secondary to pneumonia. He, unfortunately, passed away one month after admission due to complications unrelated to his oncologic condition.

## Discussion

Extramedullary involvement has been described in approximately 13-19% of myeloma patients, and most commonly affects males. It typically occurs at advanced stages as the myeloma plasma cells infiltrate locally in bone or in soft tissues. IgD secreting plasma cells are a product of somatic hypermutation of the IgD region from germinal center B cells. It is clinically characterized by a reversed light chain ratio, a very low M-spike on SPEP, and short survival time [7-8]. Prevalence of extramedullary plasmacytoma (EMP) in IgD myeloma is around 19-63% with usual sites being the chest wall, respiratory tract, gastrointestinal tract (GI) tract, skin, lymph nodes, and paraspinal areas with isolated testicular involvement being extremely rare [1,9]. The first case of IgD lambda related testicular tumor was reported by Kubonishi I, et al. in which a patient presented three months after orchiectomy with an abdominal mass and ascites. An ascitic fluid study revealed aneuploidy and 1q+, 2p+, and 14q+ - of myeloma plasma cells [9].

EMP’s in myeloma is associated with poor prognosis. Varettoni M, et al. described poor PFS (18 vs 30 months) in myeloma-related EMP diagnosed at presentation versus at follow-up [9]. The case we describe is the second report of IgD lambda myeloma manifesting as a testicular plasmacytoma on the initial presentation that we are aware of. Similar to the first cited case, our patient also underwent orchiectomy, but unlike the former, he responded excellently to combined chemotherapy and remained in remission for almost three years without relapse or complication during his clinical course. His karyotype was normal, but cytogenetic studies were positive for CCND1/IGH rearrangement and loss of TP53.

The prognosis of IgD myeloma has been described as very poor, with a median overall survival time of fewer than two years. However, in recent years following the availability of novel agents and ASCT, the prognosis has significantly improved [7-8], as exemplified by our patient’s case. Shimamoto, et al. proposed that light chain subtype and WBC were significant predictors of survival noting that individuals with white blood cells (WBC) count < 7 × 109/L and the k subtype were low risks, with a five-year overall survival (OS) of 66% [10].

It is important to note that our patient’s multiple myeloma had an excellent response to combined chemotherapy. Perhaps the poor prognosis previously described in the medical literature is related to early stage misdiagnosis as a result of a low M-spike, due to the absence of novel agents, or possibly a combination of both factors. These are merely speculations and future studies of IgD myeloma patients are necessary to determine whether the observed favorable response to treatment was related to the combination of novel agents, to factors such as WBC and light chain subtype (k/l) or to both [10].

## Conclusions

Immunoglobulin D (IgD) myeloma has been a diagnostic challenge due to an undetectable M-spike on SPEP. Malignant plasma cells can infiltrate unusual anatomic sites and can be the presenting feature of MM. This case demonstrates a relatively rare presentation and subtype of a common disease. It illustrates the response to novel therapy of this particular subtype and can guide future research in diagnosis and treatment. Furthermore, it is unknown if abnormal cytogenetics has an effect on a particular treatment or has prognostic value. Biology of the IgD subtype and its implications for treatment and prognosis, therefore, remains an important field for future exploration to improve outcomes for our patients. 

## Appendices

List of Abbreviation

Ig - Immunoglobulins

M - Monoclonal

EMP - Extramedullary plasmacytoma

PFS - Progression free survival

SPEP  -  Serum protein electrophoresis

ASCT - Autologous Stem cell transplant

FISH - Florescent in-situ hybridization

ISS - International Staging System
